# Spatial and Sequential Organization of Gaze During Facial Expression Recognition Tasks

**DOI:** 10.3390/bs16050792

**Published:** 2026-05-16

**Authors:** Alessandro De Santis, Giusi Antonia Toto, Guendalina Peconio, Laura D’Amico, Pierpaolo Limone

**Affiliations:** 1 Learning Science Institute (LSi), Department of Human Sciences, University of Foggia, 71121 Foggia, Italy; giusi.toto@unifg.it (G.A.T.); guendalina.peconio@unifg.it (G.P.); 2 Department of Human Sciences, Education and Sport, Pegaso University, 80143 Napoli, Italy; laura.damico@unipegaso.it (L.D.); pierpaolo.limone@unipegaso.it (P.L.)

**Keywords:** facial expression recognition, eye tracking, gaze organization, gaze trajectories, Markov models

## Abstract

**Background.** Facial expression recognition depends on how visual information is sampled across the face over time. Static area-of-interest (AOI) measures describe where observers look but provide limited information about the sequential organization of gaze. This study examined how gaze is organized during facial expression recognition and whether this organization remains comparable across two conditions differing in the temporal order of contextual and facial stimuli. **Methods.** Eye-tracking data were collected from 27 participants performing a facial expression recognition task. Fixations on faces were mapped onto three AOIs: Upper Facial Zone (UFZ), Central Facial Zone (CFZ), and Lower Facial Zone (LFZ). Gaze organization was examined using first- and second-order Markov models, entropy estimates, spatial repositioning measures, and a gaze stability index. **Results.** Gaze transitions showed a structured, non-random organization centered on the CFZ. In the first-order Markov model, transitions from both the UFZ and LFZ were directed primarily toward the CFZ, and within-zone transitions were also most likely in the CFZ. Entropy was lower for the CFZ than for the upper and lower regions, indicating lower transition uncertainty in the central region. The second-order model showed an influence of recent fixation history while preserving the predominance of the CFZ. Spatial repositioning varied across facial zones in both conditions. However, mixed-effects analyses showed no effect of condition on gaze stability. **Conclusions.** Facial expression recognition was associated with a pattern of exploration in which the central facial region emerged as the most likely fixation destination, with limited evidence of condition-related differences in gaze organization.

## 1. Introduction

Facial expression recognition depends on the selective sampling of visual information across the face. Rather than being distributed uniformly, visual attention tends to be allocated to specific facial regions in ways that reflect both perceptual constraints and task-related demands. For this reason, the organization of gaze during face viewing is central to how expressive information is sampled and processed.

### 1.1. Eye Movement Behavior in Facial Expression Recognition

Eye movement behavior represents a central component of facial expression recognition, as it shapes the sampling of facial information and modulates attentional and sensory processing during face viewing (Ricciardelli et al., 2016). Although facial expression recognition belongs to the broader domain of face perception, it has been suggested that the processing of facial expressions may be partly dissociable from the analysis of other facial aspects, including person identity and gaze direction (Baseler et al., 2014). Facial expression recognition (FER) is typically defined as the assignment of a facial stimulus to a specific expression category (Calvo & Nummenmaa, 2016). In this context, recognition requires observers to assign facial stimuli to predefined categories. Expression categorization and discrimination can therefore be understood as cognitive processes through which facial configurations are evaluated in terms of similarity and difference (Calvo et al., 2018). Several eye-tracking studies have therefore examined how observers sample facial information during expression recognition and which facial regions receive greater visual attention. In self-paced expression categorisation tasks, participants generally allocate longer viewing times and/or a higher number of fixations to the eye region than to the mouth or nose region (Guo, 2012).

A substantial body of research on facial expression recognition has examined how the visual system decodes facial expressions, with particular attention to the ocular strategies that support this process (Rodger et al., 2023) and to the relationship between eye movements and brain connectivity during expression recognition tasks (Zhang et al., 2022). Within these studies, the analysis of gaze behavior appears to be fundamental, both in controlled settings, such as during the viewing of stimuli presented on a screen (e.g., Wang et al., 2025), and in more ecological contexts designed to promote natural interpersonal interactions (Vehlen et al., 2021; Mayrand et al., 2023). In this framework, gaze behavior can be understood as one component of a broader set of cognitive mechanisms involved in facial expression processing, including attentional orienting and the deployment of gaze toward salient facial cues. For instance, Yang and Gamer (2025) showed that the central region of the face acts as a strong attractor for gaze deployment, reflecting an automatic tendency to orient both attention and gaze toward emotionally diagnostic facial features. This effect emerged even under very brief stimulus presentations (50 ms) and remained stable across different initial fixation positions. The findings suggest that gaze deployment may be supported by the extrafoveal processing of facial features and may reflect a holistic mode of facial expression processing. The authors also emphasized the need for further research to determine whether these results generalize to other facial expressions and viewing conditions.

Regarding attentional patterns, Alkan (2025) showed that facial expression recognition is associated with distinct distributions of visual attention during face exploration. Their findings indicate that accurate and inaccurate categorizations are characterised by different attentional allocation patterns across facial regions: whereas accurate recognition involves a broader distribution of attention across multiple diagnostically relevant areas of the face, inaccurate recognition is associated with a more restricted attentional focus, particularly on the upper facial region. These findings suggest that gaze deployment during facial expression recognition may involve more than the selection of isolated diagnostic cues, and may instead reflect a more coordinated distribution of attention across the face. This provides a useful point of departure for considering the role of holistic processing in central gaze deployment.

### 1.2. Holistic Processing and Central Gaze Deployment in FER

Guo (2012) described holistic face-processing behaviour as the scanning of key internal facial cues (i.e., the eye region, nose, and mouth) in order to extract expressive information from the face as a whole rather than from isolated facial parts. Studies on facial expressions suggest that individuals often adopt a holistic processing strategy when evaluating facial expressions (Guo, 2012; Omigbodun & Cottrell, 2013), although there is less consensus on the extent to which different expressions depend on whole-face processing. Experimental evidence indicates that holistic processing emerges particularly when there is conflict between different parts of the expression, as in the case of composite faces, whereas in the absence of such conflict, processing may be more analytic in nature (J. W. Tanaka et al., 2012). This type of processing appears to occur at very early stages of visual perception, even within 17 ms, and is associated with relatively invariant gaze patterns (Liu et al., 2020; J. Tanaka & Xu, 2018; Gregory et al., 2021; Guo & Shaw, 2015). In a study by Sun and colleagues, the use of the composite face task with “split” faces, in which the upper and lower halves were combined, revealed a congruency effect, indicating that facial expressions are processed holistically when the two halves of the face are aligned (Sun et al., 2023).

In contrast with strong holistic models of face processing, Cheng and Little (2025) suggest that, in composite faces, second-order facial features are not always integrated into a single unified percept. Rather, their findings indicate that the upper and lower halves of the face may be processed relatively independently, through a combination of serial and parallel mechanisms. These findings qualify strong holistic accounts by suggesting that, under some conditions, face processing may rely not only on configural integration, but also on more analytic mechanisms operating across separate facial regions. Central gaze deployment, therefore, should not be treated as direct evidence of mandatory holistic processing, but rather as one possible mechanism supporting coordinated sampling under specific viewing conditions. At the same time, some studies have suggested that a central fixation bias, namely the tendency to direct gaze toward the central region of the stimulus, may facilitate holistic face processing by supporting the extrafoveal sampling of multiple internal facial cues and, in turn, the configural integration of facial information (Miellet et al., 2013; Poltoratski et al., 2021; Urtado et al., 2024).

### 1.3. Contextual Effects on Attentional Patterns During Face Processing

Context provides important information for facial expression recognition and may shape both perceptual and attentional processes during face viewing. Behavioral evidence indicates that contextual information can either facilitate or hinder facial expression recognition, particularly when the face is degraded or ambiguous, and may also influence gaze behavior (Garlichs & Blank, 2024). According to Aviezer et al. (2017), facial expression recognition does not depend exclusively on facial features, but may also be modulated by contextual factors external to the expresser, such as scene information, surrounding people, and other environmental cues. Moreover, the literature has documented that context is routinely encoded during facial expression perception (Barrett & Kensinger, 2010; Mauersberger et al., 2026). Consistent with this view, Goel et al. (2024) found that inferences based on facial expressions are shaped not only by facial cues, but also by situational information, although the integration of these sources appears to be limited and variable across emotion categories and individuals. Their findings suggest that isolated facial cues are not always sufficient, and that contextual knowledge may substantially support expression interpretation.

The temporal order of contextual and facial stimuli may shape facial expression processing in different ways. When contextual information precedes the face, it may bias subsequent interpretation by providing an anticipatory frame for processing the target expression (Righart & de Gelder, 2008). When contextual information follows the face, it may instead influence the retrospective evaluation of the previously viewed expression, in line with postdictive accounts of perceptual interpretation (Cao et al., 2022). More specifically, environmental scenes with different affective properties, as well as stimuli varying in threat relevance, have been shown to modulate the processing of facial expressions (Righart & de Gelder, 2008; Öhman et al., 2001).

In addition to their semantic and affective properties, contextual stimuli may also influence the spatial allocation of attention. By introducing task-related or visuospatial constraints before target-face presentation, contextual manipulations may shape subsequent face-scanning patterns and thereby affect how facial expressions are processed (Arizpe et al., 2012). More broadly, evidence from attentional selection paradigms suggests that non-target stimuli can shape subsequent affective evaluation through inhibitory mechanisms, indicating that stimuli receiving reduced attention may nonetheless influence later processing (Gómez-Cuerva & Raymond, 2011; Goolsby et al., 2009).

### 1.4. Dynamic Approaches to Gaze Analysis in FER Tasks

Facial expression recognition tasks are commonly used to investigate how observers identify and interpret expressive facial cues under controlled conditions. These tasks typically require participants to inspect a face and classify the displayed expression, usually within a set of basic emotional categories: happiness, sadness, fear, anger, disgust, and surprise. In doing so, they elicit systematic visual exploration of diagnostically relevant facial regions. Within this framework, the Facial Action Coding System (FACS; Ekman & Friesen, 1978) provides an important reference for describing facial expressions in terms of anatomically based Action Units, thereby linking these expression categories to specific and functionally meaningful facial movements and regions relevant to expression recognition. For this reason, Facial Expression Recognition Tasks provide a suitable context for applying dynamic approaches to gaze analysis, allowing researchers to examine not only where observers look, but also how visual scanning unfolds over time. In particular, analyses based on gaze trajectories and Markov models allow researchers to examine both the spatial distribution and the temporal sequencing of fixations across the face. By modeling recurrent transitions between facial regions, these approaches provide a useful framework for identifying stable viewing patterns during facial expression recognition. A recent study applying Markov Models to eye movements showed that face learning and recognition are associated with relatively stable visual exploration patterns, broadly distinguishable into more central-focused and more eye-focused strategies (Bennetts et al., 2025). These findings suggest that observers tend to adopt consistent viewing strategies during face processing, supporting the idea that gaze organization may reflect stable underlying patterns rather than purely moment-to-moment fluctuations. Unlike static AOI-based measures, analyses of gaze trajectories retain the temporal order of fixations and thus provide a dynamic account of how visual attention is distributed across the face over time. This is particularly relevant in facial expression recognition, where information is gathered through successive fixations rather than through a single uniform sampling of the face. Recent work has emphasized the value of gaze-trajectory-based approaches for jointly capturing spatial and temporal aspects of gaze behavior, and has reported expression-related differences in gaze organization across facial expressions (Laborde et al., 2026). At the same time, Markov models provide only a partial account of this organization. Although they characterize transition probabilities between facial regions, they do not fully capture the ordered unfolding of visual exploration across successive fixations. For this reason, integrating probabilistic transition modeling with temporally ordered analyses of gaze trajectories may offer a more complete account of how facial information is sequentially sampled during facial expression recognition.

The present study applies this integrated framework to examine how gaze is organized during facial expression recognition across two procedural conditions differing in the temporal order of contextual and facial stimuli, and to assess whether this manipulation is associated with reliable differences in visual exploration.

### 1.5. The Present Study

To address this gap, the present study examined how gaze is spatially and temporally organized during facial expression recognition, with particular attention to its sequential structure. Based on the existing literature, we formulated two main hypotheses. First, we expected gaze behavior to exhibit a structured, non-random organization, characterized by a predominant allocation of fixations toward the central facial region (H1). Second, we hypothesized that the overall organization of gaze would remain broadly stable across procedural conditions such that variations in the temporal order of contextual and facial stimuli would not produce reliable changes in gaze allocation or stability across facial regions (H2).

## 2. Materials and Methods

### 2.1. Sample

Sample size was determined a priori based on previous studies, and power calculations were conducted assuming a medium effect size (Cohen’s d = 0.50), a desired statistical power of 0.80, and an alpha level of 0.05. Under these assumptions, a minimum sample size of 34 participants was required. Participants were randomly assigned to one of two experimental procedures, which differed in the type of distractor preceding the target stimulus. A total of 46 participants were recruited and distributed across the two procedures (Procedure 1: *n* = 22; Procedure 2: *n* = 24).

Data quality screening was conducted prior to analysis using predefined criteria standard in eye-tracking research, including calibration accuracy, proportion of valid gaze samples, and task compliance. Participants not meeting these criteria were excluded from further analyses. This resulted in a final sample of 27 female participants (mean age = 34 years, SD = 12.1), with 13 participants completing Procedure 1 and 14 completing Procedure 2. However, for analytical purposes, these original procedural groups were subsequently redefined according to the event immediately preceding target-face processing (see Procedure for details). All participants were residents of Southern Italy, specifically from the Apulia (Puglia) region. Regarding educational and occupational status, 61% of the sample were university students enrolled in undergraduate or graduate degree programs, while the remaining 39% were employed and concurrently enrolled in university courses. The loss of 9 participants in the first procedure and 11 in the second, together with the fact that the sample consisted exclusively of female participants and was drawn solely from the Apulia region, represents a limitation that should be taken into account when interpreting and generalizing the results.

### 2.2. Procedure

Participants were recruited through invitation letters distributed during university courses and via academic mailing lists associated with local degree programs. All participants reported normal or corrected-to-normal vision and no history of neurological or psychiatric disorders. They were naïve with respect to the aims of the study and completed the experiment individually. Written informed consent was obtained from all participants prior to participation. The study protocol was approved by the Ethical Committee of the Department of Psychology at Pegaso University (approval number 007849, 4 November 2025).

**Figure 1:**
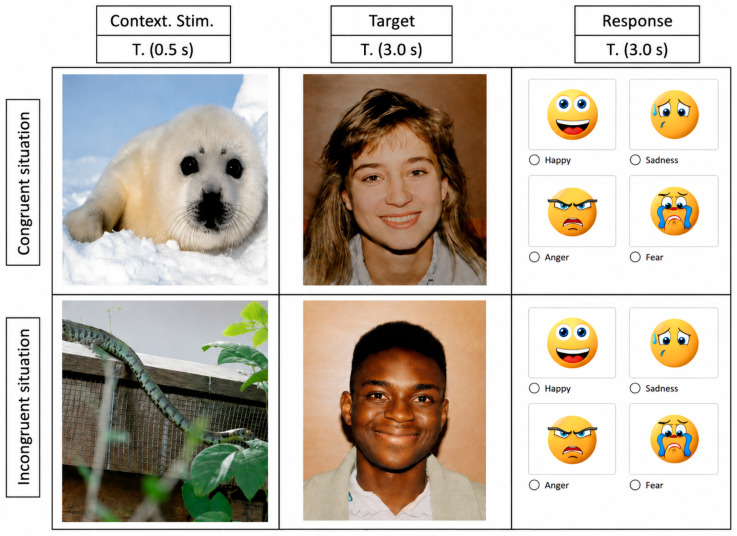
Sequence of stimuli used in the task. In the first procedure, a target face was presented for 3000 ms and was subsequently followed by a contextual stimulus for 500 ms. In the second procedure, a contextual stimulus was presented for 500 ms and was subsequently followed by a target face for 3000 ms. In both procedures, the response stimulus screen was presented last for 5000 ms. The two procedures were designed to manipulate event order and examine whether gaze deployment toward facial expressions varied as a function of sequential context.

Participants were randomly assigned to one of two eye-tracking procedures that differed in the temporal placement of the contextual stimulus relative to the target face. In the first procedure (post-target context condition), each trial began with a facial target stimulus presented for 3000 ms, followed by a contextual stimulus presented for 500 ms. A response screen was then displayed for up to 5000 ms. In the second procedure (pre-target context condition), the order was reversed: each trial began with a contextual stimulus (500 ms), followed by the facial target stimulus (3000 ms), and then by a response screen displayed for up to 5000 ms (see Figure 1). All other experimental parameters, including stimulus duration, response window, and task instructions, were identical across procedures. Responses were provided via a key press (space bar) following target presentation.

For the purposes of the present study, however, the two procedural groups were redefined according to the task event immediately preceding target-face onset. Under this analytical framework, the relevant pre-target event was the response screen from the previous trial in Procedure 1 and the contextual stimulus presented within the current trial in Procedure 2. The temporal sequence of stimuli allowed us to impose spatial constraints on gaze between successive target-face presentations. In Procedure 1, gaze was modulated by the preceding response screen, which constrained eye position through gaze-based response selection and the use of the space bar to confirm the participant’s choice. In Procedure 2, gaze before target-face presentation was constrained by contextual stimuli.

### 2.3. Stimuli Selection

Contextual stimuli consisted of images selected from the OASIS database (Kurdi et al., 2017), an open-access and validated affective image set commonly used in emotional priming research. Although typically employed in priming paradigms, in the present study, these stimuli were used as contextual distractors preceding the target, allowing manipulation of the affective context without directly targeting priming effects. The selected stimuli were grouped into two main categories: animals and environmental scenes. In both cases, images were chosen to represent varying levels of threat relevance (e.g., dog vs. snake for animals; snowy landscape vs. polluted industrial area for environmental scenes). Emotional categories for facial expressions were defined based on the DANVA-2 stimulus set (Diagnostic Analysis of Nonverbal Accuracy; Nowicki & Duke, 1994), a standardized and widely used tool in emotion perception research. This set includes four target emotions: happiness, sadness, anger, and fear, which were selected in line with the task-accuracy-based stimulus selection framework proposed by Nowicki and Duke (1994) for the categorization of expressive faces. The response screen consisted of four emojis corresponding to the four target expressions defined in the DANVA framework, allowing participants to categorize each facial stimulus. Contextual and target stimuli were combined to create emotionally congruent and incongruent conditions. In congruent trials, the affective valence of the contextual stimulus matched the facial expression of the target, whereas in incongruent trials, the contextual information and the expressive face conveyed mismatched emotional content. Importantly, in the present study, neither contextual stimuli nor the response screen were treated as primary affective manipulations or as measures of emotion recognition accuracy. Rather, they were considered task events capable of modulating gaze and defining different visuospatial states prior to target-face processing. More specifically, the event immediately preceding the target differed across procedures: the contextual stimulus in Procedure 2 and the response screen from the preceding trial in Procedure 1.

### 2.4. Eye Tracker Procedure Set-Up

Eye movements were recorded using a remote Tobii eye-tracker controlled via Tobii Pro Lab software 25.7. Participants were seated approximately 70 cm from a 23-inch LCD monitor (1920 × 1080 resolution, 60 Hz refresh rate) in a quiet, dimly lit room and were instructed to maintain a stable head position throughout the task. Stimuli were presented centrally on the screen against a uniform background. Prior to the experimental session, all participants completed a standard 9-point calibration procedure. Calibration accuracy was visually inspected and repeated when necessary until reliable tracking quality was achieved for both eyes. Following calibration, participants completed a face expression recognition task across two experimental procedures. The entire procedure lasted approximately 10 min.

### 2.5. Definition of Areas of Interest

Target areas of interest (AOIs) were manually defined in Tobii Pro Lab based on anatomically anchored facial landmarks to ensure consistency across stimuli. Boundaries were delineated using structural markers including the supraorbital ridge, the subnasale, and the menton. Accordingly, the Upper Facial Zone extended from the forehead to the supraorbital ridge, the Central Facial Zone extended from the supraorbital ridge to the subnasale, and the Lower Facial Zone extended from the subnasale to the chin. This operational segmentation was based on a topographic-functional grouping of Action Units derived from the Facial Action Coding System (FACS; Ekman & Friesen, 1978), rather than on an official zonal subdivision within FACS. Specifically, the Upper Facial Zone comprised Action Units primarily associated with brow and upper periorbital movements (AU1, AU2, AU4, AU5, AU7); the Central Facial Zone comprised mid-face activity, including cheek, nasal, and upper-lip related movements (AU6, AU9, AU10, AU11); and the Lower Facial Zone comprised Action Units related to the mouth, lips, jaw, and chin (AU12, AU14, AU15, AU16, AU17, AU18, AU20, AU22, AU23, AU24, AU25, AU26, AU27, AU28). Importantly, this region-based segmentation is also supported by evidence showing that emotion-relevant information is distributed across diagnostic facial features, with preferential attentional orienting toward functionally relevant regions such as the eye region, with attentional prioritization varying as a function of the emotional expression, reflecting the functional specialization of facial regions rather than isolated feature processing (Yang & Gamer, 2025). Accordingly, AOIs were defined at a regional level rather than at the level of individual Action Units to preserve anatomical coherence across heterogeneous face stimuli and to capture region-based visual processing.

### 2.6. Data Administration and Analytical Strategy

Data were analyzed using MATLAB (R2024b The MathWorks, Inc., Natick, MA, USA) and R (version 4.4.1.; R Core Team, YEAR; R Foundation for Statistical Computing, Vienna, Austria). Data were processed and analyzed following two complementary procedures, each designed to address a distinct theoretical question regarding facial expression processing.

Before analysis, gaze data were preprocessed by converting spatial coordinates into numeric format and removing observations with missing values. Only valid fixations were retained for subsequent analyses. Data were then segmented by participant, item, and event. Each fixation was assigned to one of three predefined facial areas of interest previously mentioned, defined at the item level.

To test the first hypothesis, Markov modeling was applied to the sequences of AOIs for each participant, providing a formal framework to assess whether gaze transitions followed a structured, non-random organization. Transition matrices were estimated for both first-order (AOI_t−1_ → AOI_t_) and second-order models (AOI_t−2_, AOI_t−1_ → AOI_t_). From these matrices, transition probabilities, state-wise entropy, and global normalized entropy were computed. Differences between first- and second-order entropy were assessed using Wilcoxon signed-rank tests, complemented by bootstrap estimation of the entropy difference (H_1_ − H_2_). First-order Markov transition probabilities were calculated as the conditional probability of transitioning from one AOI to another based on the immediately preceding fixation, $\left(P\left(X_{t}=j|X_{t-1}=i\right)\right)$, where (X_t_) denotes the AOI at time (t), and (i) and (j) represent the origin and destination AOIs, respectively. Second-order Markov transition probabilities were computed as the conditional probability of transitioning to a given AOI based on the two preceding fixations $\left(P\left(X_{t}=k|X_{t-2}=i,X_{t-1}=j\right)\right)$, where (i), (j), and (k) denote consecutive AOI states in the fixation sequence.

An anchor-based transition analysis was conducted across two conditions by computing Euclidean distances between the final fixation of the preceding event, used as the anchor, and the first fixation on the subsequent target. The preceding event served exclusively as a spatial reference point to define the starting location of the transition.

Euclidean distance was calculated as:

$$d=\sqrt{{\left(x_{2}-x_{1}\right)}^{2}+{\left(y_{2}-y_{1}\right)}^{2}}$$

Differences across AOIs were assessed using Kruskal–Wallis tests, followed by pairwise Wilcoxon comparisons with Holm correction. Following the first-saccade transition analysis, path length was calculated as the sum of Euclidean distances between consecutive fixation points within each AOI:

$$\mathrm{PathLength}=\sum_{i=1}^{n-1}\sqrt{{\left(x_{i+1}-x_{i}\right)}^{2}+{\left(y_{i+1}-y_{i}\right)}^{2}}$$

**Figure 2:**
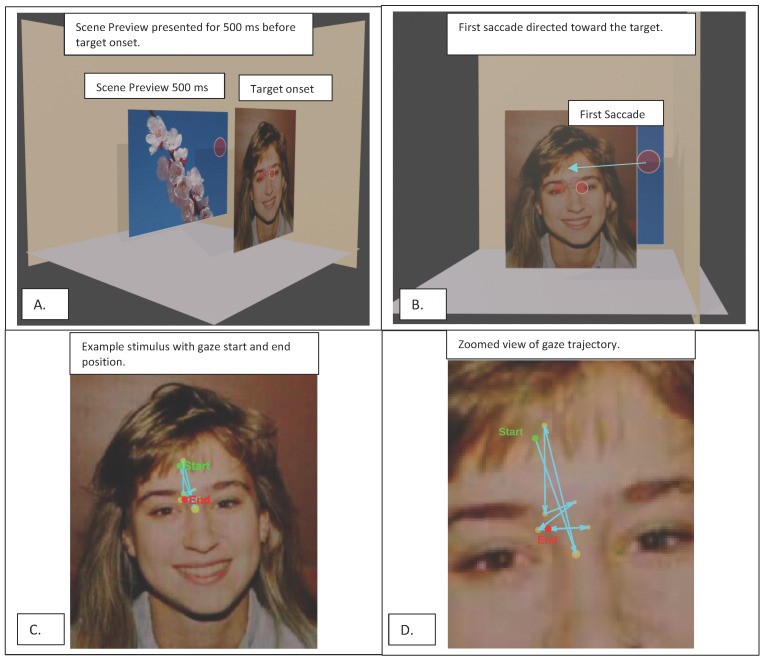
Illustration of the experimental sequence and gaze trajectory computation. (**A**) Scene preview presented for 500 ms before target onset. (**B**) Target onset and first saccade directed toward the peripheral target. (**C**) Example stimulus showing gaze start and end positions. (**D**) Zoomed view of the gaze trajectory, highlighting the initial fixation point, the endpoint of the first saccade, and the computed transition path.

The sequence of events and the computation of the first saccadic transition are illustrated in Figure 2. The figure provides an overview of the experimental timeline and the spatial definition of gaze transitions from the preceding event to the target.

**Figure 3:**
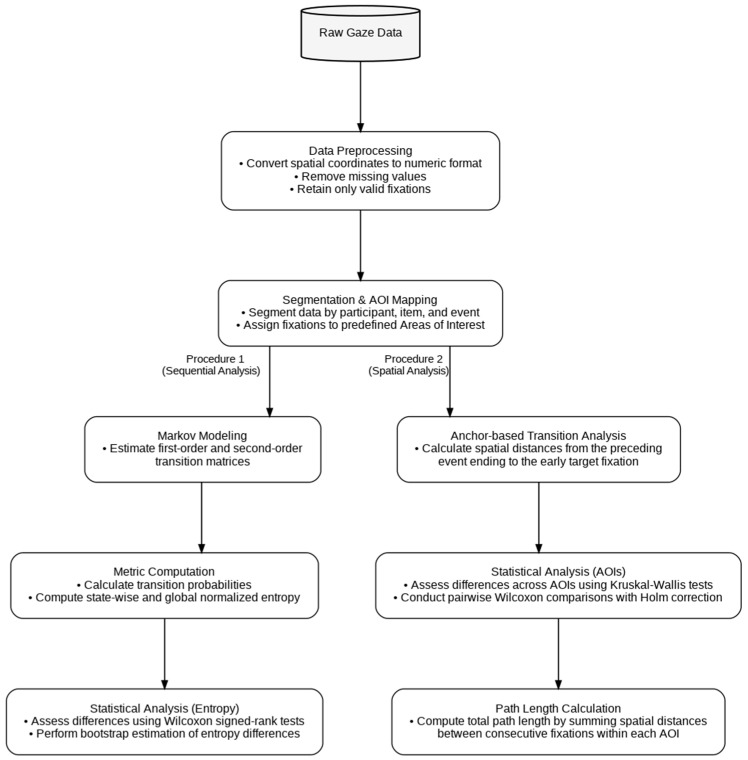
Overview of the gaze analysis pipeline, including preprocessing, AOI mapping, and two parallel procedures: sequential modeling of gaze transitions and spatial analysis of fixation distances and AOI-based metrics.

Gaze stability was quantified as $\left(\mathrm{Stability}=\frac{\mathrm{Total}\;\mathrm{Duration}}{\mathrm{PathLength}}\right)$, where path length was defined as the cumulative Euclidean distance between consecutive fixation points. Data were then reorganized into long format (Participant × AOI) for statistical analysis. Stability values were analyzed using linear mixed-effects models (LMMs), including models with AOI as a fixed effect and participant as a random intercept (Stability ~ AOI + (1|Participant)), as well as models including EventType and its interaction with AOI (Stability ~ AOI + EventType_1/2_ + (1|Participant)). Model effects were evaluated using analysis of variance (ANOVA) on the fitted mixed models. A path-based visualization approach was used to reconstruct fixation sequences for a single item and to map gaze trajectories across participants, preserving the temporal order of fixations. Inter-participant concordance of gaze patterns was assessed using Kendall’s coefficient of concordance (W). The coefficient was computed as $\left(W=\frac{12S}{m^{2}\left(n^{3}-n\right)}\right)$, where $\left(S=\sum_{i=1}^{n}{\left(R_{i}-\bar{R}\right)}^{2}\right)$, (m) is the number of participants, (n) is the number of spatial units, (R_i_) is the sum of ranks for unit (i), and $\left(\bar{R}\right)$ is the mean of these sums. The following diagram (Figure 3) illustrates the analysis pipeline, structured into two complementary procedures: a sequential analysis based on Markov modeling of gaze transitions, and a spatial analysis focusing on anchor-based transition distances and AOI-level comparisons.

## 3. Results

### 3.1. Behavioral Performance in the FER Task

As a descriptive index of task performance, participants recognized facial expressions with an overall accuracy of 60.2%, above the nominal chance level of 25%. Accuracy varied across emotion categories, with the highest performance for happiness (79.6%), followed by sadness (65.6%), anger (50.0%), and fear (45.6%). Mean response time during the response phase was 3.82 s (SD = 0.74 s). These descriptive results indicate that participants engaged with the facial expression recognition task at above-chance levels.

### 3.2. Sequential Analyses of Gaze Transitions

**Figure 4:**
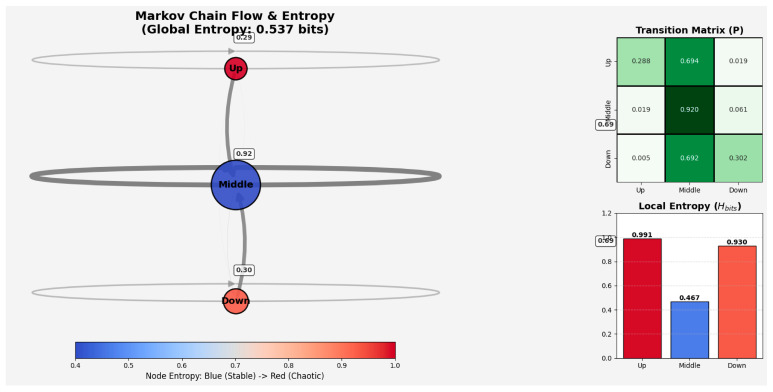
First-order Markov transition structure and entropy across facial zones. Transition probabilities were concentrated around the Central Facial Zone, with frequent shifts from the Upper and Lower zones to the Central zone. Local entropy was lower for the Central Facial Zone than for the Upper and Lower zones, indicating lower transition uncertainty in the central region.

To examine the sequential organization of gaze across facial regions, we conducted a first-order Markov analysis of fixation transitions among the Upper, Central, and Lower Facial Zones. The transition matrix showed that gaze shifts were more frequently directed toward the Central Facial Zone than between the upper and lower regions, with relatively frequent transitions from both the Upper and Lower zones to the Central zone (0.694 and 0.692, respectively), infrequent direct transitions between the Upper and Lower zones, and a high probability of successive fixations within the Central Facial Zone (0.920). Normalized entropy (H_norm), used here as an index of transition uncertainty, showed lower uncertainty for transitions originating from the Central Facial Zone (H_norm = 0.295) than for those originating from the Upper (H_norm = 0.625) and Lower Facial Zones (H_norm = 0.587), indicating more predictable fixation shifts from the central region. At the global level, normalized entropy was 0.339 (H_bits = 0.537), suggesting that fixation transitions across facial zones were not uniformly distributed overall. Figure 4 summarizes the first-order Markov and entropy results. Transition probabilities were concentrated around the Central Facial Zone, with frequent shifts from the Upper and Lower zones to the Central zone and lower transition uncertainty for the Central than for the other facial zones.

To further examine whether fixation transitions depended on recent fixation history, we conducted a second-order Markov analysis estimating transition probabilities as a function of the two preceding fixation states. Across most state combinations, the Central Facial Zone remained the most likely destination, particularly when the immediately preceding fixation was also located in the Central Facial Zone (e.g., Upper-Central → Central = 0.902; Central-Central → Central = 0.926; Lower-Central → Central = 0.830). By contrast, transitions to the Upper Facial Zone were relatively infrequent when recent fixation history involved the Lower Facial Zone, whereas persistence in the Lower Facial Zone was observed mainly when both preceding fixations were also located in the Lower Facial Zone (Lower-Lower → Lower = 0.535). Overall, these conditional probabilities suggest that fixation transitions were influenced by recent fixation dynamics while remaining predominantly centered on the Central Facial Zone.

Entropy estimates derived from the second-order transition matrix suggested a modest influence of recent fixation history. In particular, normalized entropy was lower when the immediately preceding fixation was located in the Central Facial Zone (e.g., Upper-Central = 0.321; Central-Central = 0.273; Lower-Central = 0.414), indicating lower transition uncertainty for sequences ending in the central region. By contrast, higher entropy values were observed for several sequences ending in the Upper or Lower Facial Zones, suggesting comparatively greater variability in subsequent fixation shifts. At the global level, normalized entropy for the second-order transition model was 0.319 (H_bits = 0.505), indicating a relatively low degree of transition uncertainty overall.

**Figure 5:**
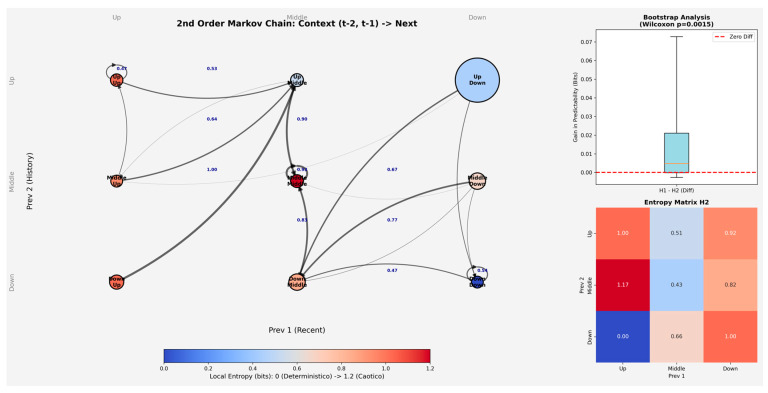
Second-order Markov transition structure across facial zones. Transition probabilities varied as a function of the two preceding fixation states while remaining predominantly centered on the Central Facial Zone. Node color reflects local entropy, with lower values indicating lower transition uncertainty.

Figure 5 summarizes the main results of the second-order Markov analysis. Transition probabilities varied as a function of the two preceding fixation states while still showing a predominance of transitions toward the Central Facial Zone and relatively low overall transition uncertainty. Although transition sequences were constructed within each Participant × Item series and participant-level entropy estimates were compared between first- and second-order models, the main Markov transition matrices were estimated at the aggregate level and therefore do not directly model inter-individual variability in gaze strategies.

To compare the first- and second-order models, we examined the bootstrap distribution of the entropy difference (H1 − H2). The estimated median difference was 0.0048, with a percentile-based 95% interval ranging from −0.0018 to 0.0668. Although the interval included zero, the Wilcoxon signed-rank test indicated that the differences were generally greater than zero (V = 281, *p* = 0.0015). Overall, this pattern suggests a small reduction in entropy in the second-order model.

### 3.3. Spatial Repositioning Analyses

To examine spatial repositioning across successive task events, we adopted an anchor-based approach, defining the last fixation on the preceding response screen as the spatial reference point. Euclidean distance was then calculated between this anchor and the first available fixation on the subsequent target face. In Condition 1, this distance varied across facial zones (Kruskal–Wallis χ^2^ = 11.06, *p* = 0.004 (ε^2^ = 0.075)), with a significant pairwise Wilcoxon difference between the Upper and Central Facial Zones (*p* = 0.004; r = 0.26). In Condition 2, the same measure also differed across facial zones (Kruskal–Wallis χ^2^ = 4.33, *p* = 0.037 (ε^2^ = 0.023)), with a significant pairwise Wilcoxon difference between the Central and Lower Facial Zones (*p* = 0.038; r = 0.17). Overall, the results suggest that spatial repositioning across trials varied as a function of the facial zone reached on the target stimulus. This analysis should be interpreted as indexing between-event spatial re-centering rather than early attentional orienting.

**Figure 6:**
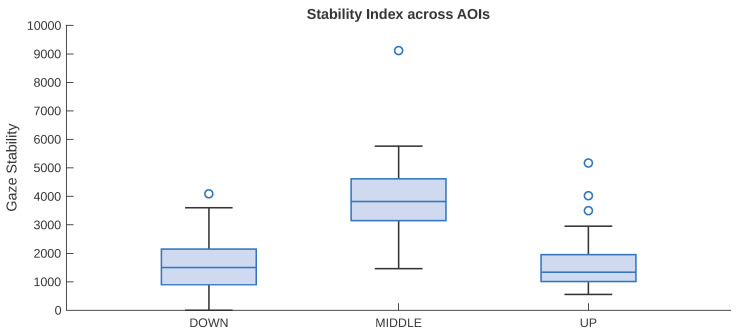
Gaze stability index across facial zones. Boxplots show the distribution of the stability index in the Upper, Central, and Lower Facial Zones. Higher values indicate greater gaze stability within the corresponding facial region.

After reconstructing gaze trajectories, we computed for each participant and facial zone a gaze stability index defined as the ratio between total fixation duration and path length. These values were then entered into a linear mixed-effects model, with AOI as a fixed effect and participant as a random intercept. The model showed a significant effect of AOI on gaze stability (F(2, 78) = 36.15, *p* < 0.001). Relative to the Lower Facial Zone, stability was significantly higher in the Central Facial Zone (b = 2522.7, SE = 330.57, t = 7.63, *p* < 0.001), whereas the Upper Facial Zone did not differ significantly from the Lower Facial Zone (b = 187.84, SE = 330.57, t = 0.57, *p* = 0.572). Overall, this pattern indicates that gaze was more stable in the Central Facial Zone than in the peripheral facial zones. Figure 6 summarizes the distribution of the gaze stability index across the Upper, Central, and Lower Facial Zones. As shown, stability values were higher in the Central Facial Zone than in the peripheral zones, consistent with the mixed-model results.

We further tested whether gaze stability varied as a function of procedural condition (EventType), facial zone (AOI), and their interaction using a linear mixed-effects model with participant as a random intercept. The main effect of EventType (F(1, 966) = 0.03, *p* = 0.858), the main effect of AOI (F(2, 966) = 1.73, *p* = 0.178), and their interaction (F(2, 966) = 1.06, *p* = 0.348) did not reach significance. Overall, there was no evidence that the event preceding target-face processing significantly affected gaze stability, either overall or as a function of facial zone.

**Figure 7:**
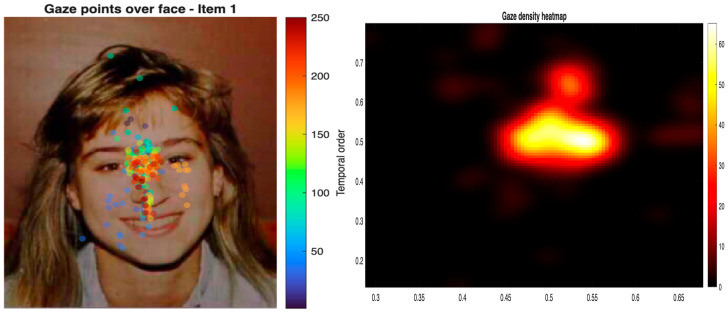
Path-based visualization of gaze observations and overall gaze density. The left panel shows temporally ordered gaze points for Item 1 across participants, with colors indicating fixation order within participants. The right panel shows the overall heatmap of gaze density across the facial display.

Finally, as a descriptive complement to the quantitative analyses, Figure 7 presents a path-based visualization of gaze observations for Item 1 across participants, in which each fixation is displayed according to its temporal order within participants, together with the overall gaze density heatmap. Both panels show a concentration of fixation activity in the central facial region.

## 4. Discussion

The present study examined how gaze is spatially and sequentially organized during facial expression recognition by combining probabilistic, entropy-based, and spatiotemporal analyses of gaze trajectories across two procedural conditions. In line with our first hypothesis, gaze behavior appeared to be structured rather than random, with fixations tending to concentrate on the central facial region. This pattern emerged in the first-order Markov analysis, where transitions from both the Upper and Lower Facial Zones were directed primarily toward the Central region, and where within-zone transitions were also most likely in the Central Facial Zone. Entropy estimates were likewise lower for the Central region than for the upper and lower regions, indicating lower transition uncertainty and a more regular fixation pattern around the center of the face. The second-order Markov analysis extended this pattern by showing that fixation transitions were influenced, to some extent, by recent fixation history. Even when the two preceding fixation states were taken into account, however, the Central Facial Zone remained the most likely destination across most state combinations. The second-order model also yielded a modest reduction in entropy relative to the first-order model, suggesting that gaze organization was not fully captured by immediate transitions alone. At the same time, the limited size of this reduction indicates that recent fixation history refined, rather than substantially altered, the broader transition structure. This pattern is in line with previous work emphasizing central gaze deployment during face viewing, as well as with accounts suggesting that facial information is often sampled in a coordinated rather than purely feature-isolated manner (Yang & Gamer, 2025; Guo, 2012). In this sense, the present findings are compatible with the literature indicating that viewers do not distribute fixations uniformly across the face, but instead tend to show structured sampling patterns that give relative priority to central facial information.

As for the second hypothesis, the spatial repositioning analysis provided a complementary perspective on whether changing the temporal order of contextual and facial stimuli was associated with differences in gaze organization across conditions. Euclidean distance measures showed that cross-event spatial repositioning varied as a function of the facial zone reached on the target stimulus in both procedural conditions. In the condition in which the target face was preceded by a contextual stimulus, this pattern suggests that gaze repositioning toward the face was not spatially uniform across facial zones. At the same time, given the 500 ms duration of the contextual stimulus, this effect should not be interpreted as reflecting early attentional alignment alone, since additional cognitive or affective processes may also have contributed to the observed pattern. Moreover, these results should be interpreted cautiously. Rather than indexing early attentional orienting in a strict sense, this measure captured the spatial repositioning of gaze between successive task events. For this reason, the distance results are better understood as evidence that between-event spatial realignment was not identical across facial zones, rather than as direct evidence about the initial spatial distribution of gaze on the target face. Importantly, gaze stability did not vary significantly as a function of procedural condition, nor did this effect differ across facial zones in the mixed-effects analyses. This suggests that, in the present dataset, the temporal order of contextual and facial stimuli was not associated with reliable differences in gaze stability. In relation to previous studies on contextual influences in face processing, this result does not imply that contextual information is irrelevant to facial expression recognition. Rather, it indicates that the procedural manipulation adopted here did not produce detectable changes in this component of gaze behavior. This interpretation is not necessarily at odds with the literature on contextual influences in face processing, which suggests that such effects may depend on task demands (Aguado et al., 2019), stimulus timing (Irwantoro et al., 2023), and the specific component of processing being examined (Goel et al., 2024). The convergence of results across analyses should therefore be interpreted cautiously. Although it points to some regularity in gaze deployment, the present design does not allow us to conclude that this regularity is specific to emotional face processing, since gaze allocation may also be influenced by more general visuospatial or task-related factors. In this respect, the present findings do not provide strong evidence for context-related changes in gaze organization, and instead suggest some regularity in gaze allocation across conditions.

From this perspective, the present results characterize facial expression recognition as an ordered pattern of visual exploration unfolding over time, rather than as a static distribution of isolated fixations across facial cues. Markov models captured transition regularities and uncertainty across facial regions, whereas trajectory-based analyses preserved the temporal progression of gaze.

## 5. Limitations and Future Directions

Several limitations of the present study should be acknowledged. First, the use of static stimuli presented on a monitor may have contributed to the well-documented central fixation bias observed in eye-tracking research, potentially limiting the generalizability of the findings to more ecological viewing conditions. In this sense, the predominance of central gaze observed in the present study may partly reflect a general viewing tendency rather than a mechanism specific to facial expression recognition. In addition, the use of different stimulus sets (OASIS and DANVA-2) across event types may have introduced variability in low-level visual features, such as luminance, contrast, or visual complexity. Future studies should consider controlling these properties more systematically or adopting more homogeneous stimulus sources. Furthermore, the 500 ms presentation of contextual stimuli is likely sufficient to engage not only early attentional alignment but also additional cognitive and affective processes, making it difficult to isolate strictly early contextual influences. More generally, although the observed gaze patterns are compatible with accounts emphasizing coordinated or relatively stable visual exploration, the present design does not directly test holistic face processing. As such, the findings should not be interpreted as direct evidence of holistic mechanisms. Similarly, the contextual manipulation was operationalized through procedural sequencing rather than through a direct test of predictive or postdictive mechanisms, and conclusions regarding contextual modulation should therefore remain limited to event-order effects. Methodologically, the distance-based analysis indexed cross-event spatial repositioning rather than the precise centrality of the initial fixation on the face, and should thus be interpreted with caution.

An additional limitation concerns the discretization of fixations into predefined AOIs, which may have reduced the spatial resolution of gaze data and obscured fine-grained variations in scanpaths. Although the present AOI-based approach was useful for testing region-level hypotheses, it may not fully preserve the continuous structure of gaze trajectories. Future studies should complement this strategy with continuous-space trajectory models to better capture spatially nuanced patterns of visual exploration. Finally, although task performance was above chance, the present study did not examine how gaze behavior varied as a function of recognition accuracy, emotion category, contextual stimulus type, or congruency. Future research should address these dimensions to better characterize the relationship between gaze dynamics, task performance, and emotional processing.

## 6. Conclusions

The present study examined how gaze is spatially and sequentially organized during facial expression recognition by combining probabilistic and trajectory-based analyses across two procedural conditions differing in the temporal order of contextual and facial stimuli. The findings indicate that gaze behavior was not randomly distributed across the face, but followed a structured pattern characterized by a predominant orientation toward the central facial region. This pattern emerged across first- and second-order transition analyses and was accompanied by lower entropy in the central region, suggesting a more regular organization of gaze around the center of the face. At the same time, variation in the temporal order of contextual and facial stimuli was not associated with reliable changes in gaze stability across conditions. Taken together, these findings suggest that facial expression recognition involves an ordered pattern of visual exploration that remains relatively consistent across the procedural conditions examined here. More broadly, the study supports the usefulness of combining probabilistic transition models with temporally ordered analyses of gaze trajectories to characterize how facial information is sampled over time during expression recognition.

## Data Availability

The datasets generated and analyzed during the current study are available in the Open Science Framework (OSF) repository (Supplementary Materials) and will be made publicly accessible upon acceptance of the manuscript.

## Supplementary Materials

Supplementary Materials are available in the Open Science Framework (OSF) repository and will be made publicly accessible upon acceptance of the manuscript. https://osf.io/hngr2/overview?view_only=5ca13cdfd8534a97b0a1ba1c01270c68 (accessed on 11 May 2026).
